# Serum ferritin and the risk of short-term mortality in critically ill patients with chronic heart failure: a retrospective cohort study

**DOI:** 10.3389/fphys.2023.1148891

**Published:** 2023-07-13

**Authors:** Zijing Zhou, Deyi Yang, Chan Li, Ting Wu, Ruizheng Shi

**Affiliations:** ^1^ Department of Cardiovascular Medicine, The Third Xiangya Hospital, Central South University, Changsha, Hunan, China; ^2^ National Clinical Research Center for Digestive Diseases, Beijing Digestive Disease Center, Department of Gastroenterology, Beijing Friendship Hospital, Capital Medical University, Beijing, China; ^3^ Department of Cardiovascular Medicine, Xiangya Hospital, Central South University, Changsha, Hunan, China

**Keywords:** serum ferritin, iron stores, inflammation, short-term mortality, chronic heart failure, prognostic marker

## Abstract

**Background:** Serum ferritin levels are associated with a higher risk of incident heart failure (HF). Whether serum ferritin levels, either increased or decreased, predict the risk of mortality in individuals with chronic heart failure (CHF) remains unknown.

**Objectives:** This study aimed to clarify the potential predictive significance of serum ferritin levels in assessing the short-term mortality in critically ill patients with chronic heart failure (CHF).

**Methods:** Critically ill patients with CHF were identified from the Multiparameter Intelligent Monitoring in Intensive Care III and IV (MIMIC III and IV) databases. Linear and logistic regression models and Cox proportional hazards models were applied to assess the associations between serum ferritin and survival.

**Results:** A total of 1,739 and 2,322 patients with CHF identified from the MIMIC III and IV databases, respectively, fulfilled the inclusion criteria. In the MIMIC III group, compared with the reference group (serum ferritin ≥70 and <500 ng/mL), serum ferritin ≥1000 ng/mL was a significant predictor of 28-day (odds ratio [OR], 1.76; 95% confidence interval [CI], 1.14–2.72) and 90-day mortality (OR, 1.64; 95% CI, 1.13–2.39). The results from the Cox regression and Kaplan–Meier curves revealed similar results. In the MIMIC IV group, serum ferritin ≥1000 ng/mL was a significant predictor of in-hospital (OR, 1.70; 95% CI, 1.18–2.46), 28-day (OR, 1.83; 95% CI, 1.24–2.69), and 90-day mortality (OR, 1.57; 95% CI, 1.11–2.22) after adjusting for confounding factors.

**Conclusion:** High ferritin levels (≥1000 ng/mL) were associated with increased short-term mortality in critically ill patients with CHF, indicating that serum ferritin may serve as a useful prognostic marker for CHF.

## Introduction

Ferritin, an iron-binding protein, comprises two isoforms of polypeptide chains, light-chain (L) and heavy-chain (H) isoforms, which cannot be distinguished by detection assays. Iron-responsive proteins partly regulate ferritin synthesis by binding to iron-responsive elements in ferritin messenger ribonucleic acid. However, this binding can be prevented by increased iron levels, leading to the facilitation of the translation of ferritin. Conversely, iron deficiency inhibits ferritin synthesis (MUCKENTHALER et al., 2008). Although an array of measures for evaluating iron levels exists, serum ferritin is an important and commonly deployed test applied in clinical and public health settings (MEI et al., 2005). Iron deficiency is associated with poor quality of life among patients with heart failure (HF) and increases the risk of hospitalization and mortality (JANKOWSKA et al., 2010; JANKOWSKA et al., 2013; KLIP et al., 2013; ENJUANES et al., 2014). Nevertheless, excessive iron accumulation in the body, a condition termed iron overload, is toxic because elemental iron participates in redox reactions and in the production of free radicals, which causes organ damage (FANG et al., 2018). Iron overload has been shown to aggravate cardiac fibrosis in angiotensin II-infused rats (ISHIZAKA et al., 2002) and to cause vascular dysfunction and the progression of atherosclerotic lesions (VINCHI et al., 2020). Excessive iron has been associated with cardiomyopathy and a higher risk of HF (KREMASTINOS and FARMAKIS, 2011). Therefore, regardless of whether a deficiency or overload, iron stores significantly contribute to cardiovascular diseases.

Moreover, serum ferritin is an acute-phase protein that reflects the degree of acute and chronic inflammation; multiple sources of evidence suggest an important role of ferritin in chronic inflammatory diseases (AROSIO et al., 2017; CHO et al., 2017; NAMASTE et al., 2017). In inflammatory conditions, ferritin synthesis is upregulated by cytokines, through a process independent of iron homeostasis (GABAY and KUSHNER, 1999). Elevated proinflammatory cytokines and inflammatory biomarkers, such as tumor necrosis factor and interleukin-1 or -6, have been observed in chronic HF (CHF), supporting the hypothesis that inflammation contributes to CHF (MANN, 2015; D'ELIA et al., 2015). Therefore, serum ferritin levels may be closely associated with CHF in terms of both iron homeostasis and inflammation.

Studies have reported that women with increased ferritin levels have a higher risk of developing HF and that elevated ferritin levels are closely associated with the incidence of type 2 diabetes (KLIP et al., 2017; SUÁREZ-ORTEGÓN et al., 2022). SILVESTRE et al. (2017) pointed out that both low and high ferritin levels were independent risk factors for incident HF in the general population. However, whether serum ferritin levels, either increased or decreased, predict the risk of mortality in individuals with CHF remains unknown. Additionally, in clinical practice, serum ferritin levels are not routinely measured in patients with HF. Therefore, this study evaluated the relationship between the serum ferritin level and the risk of short-term mortality in critically ill patients with CHF and aimed to provide evidence for physicians to make better decisions regarding therapeutic approaches and to pay attention to the clinical importance of ferritin levels.

## Materials and methods

This retrospective cohort study was reported according to the STrengthening the Reporting of OBservational studies in Epidemiology (STROBE). The project was approved by the Medical Ethics Committee for Clinical Research, Xiangya Hospital, Central South University (2022111057). All procedures were followed in accordance with the ethical standards of the responsible committee on human experimentation (institutional or regional) and with the Declaration of Helsinki 1975.

### Source of data

The retrospective cohort study was based on the Multiparameter Intelligent Monitoring in Intensive Care III and IV (MIMIC III and IV) databases, which are large US-based critical care databases. The MIMIC III database integrated comprehensive and high-quality data of patients admitted to the intensive care unit (ICU) of Beth Israel Deaconess Medical Center between 2001 and 2012, while the MIMIC IV database contained ICU patients from the same center between 2008 and 2019. After completing the training course, one author (TW) was able to access the database and was responsible for data extraction (certification number 41115067).

### Subject selection

All patients in the database with the first ICU admission were selected. The inclusion criteria were as follows: 1) patients who were diagnosed with CHF using International Classification of Diseases, Ninth and Tenth Revision (ICD-9 and -10) codes and 2) patients who had data on ferritin and iron. We excluded patients aged <18 years and those admitted to the ICU for <24 h.

### Variable extraction

Data were extracted using PostgreSQL, and the following variable data were extracted: age, sex, weight, height, heart rate (HR), mean blood pressure (MBP), and the Sequential Organ Failure Assessment (SOFA) score. The SOFA score was calculated within the first 24 h after the ICU admission. Comorbidities including hypertension, diabetes, atrial fibrillation (AF), acute kidney injury (AKI), acute myocardial infarction (AMI), chronic kidney disease (CKD), chronic obstructive pulmonary disease (COPD), and prior-AMI were also collected for analysis. Laboratory variables including creatine kinase (CK), serum creatinine (Scr), blood urea nitrogen (BUN), glucose, potassium, calcium, magnesium, sodium, white blood cell (WBC), red blood cell (RBC), hemoglobin (Hb), platelet (PLT) count, ferritin, and iron were recorded. For patients with multiple measurements, the highest daily value was included for analysis. Prescriptions including angiotensin-converting enzyme inhibitor/angiotonin receptor blocker (ACEI/ARB), β-blocker, and diuretics within 48 h after the hospital admission were analyzed.

### Outcomes

The start date for the follow-up was the date of hospital admission. All patients were observed for at least 90 days. The primary outcome was 90-day mortality. Secondary outcomes were in-hospital and 28-day mortality. In addition, 1- and 5-year mortality, the ICU length of stay (ICU-LOS), and the rate of readmission (30 days and 1 year) were also explored.

### Statistical analysis

Continuous data were presented as mean ± standard deviation or median and interquartile range (IQR), while the categorical variables were presented by the total number and frequency. The comparison between groups was performed using the X^2^ test or Fisher’s exact test for categorical variables and Student’s *t*-test or Wilcoxon rank-sum test for continuous variables. All screening variables contained less than 10% missing values. Missing values for continuous variables were imputed using the mean or median. Participants were divided into four groups based on ferritin levels (<70; ≥70 and <500; ≥500 and <1000; ≥1000 ng/mL), and group 2 (≥70 and <500 ng/mL) was selected as the reference group.

The relationship between serum ferritin levels and the risk of short-term mortality was determined using multivariate logistic regression analysis, Kaplan–Meier (K–M) curves, and Cox proportional hazards models. Baseline variables that showed a univariate relationship with the outcome (*p* <0.05) were entered into a multivariate model. In model 1, we adjusted for age, gender, body mass index (BMI), SOFA score, and MBP. In model 2, covariates were adjusted for age, gender, BMI, SOFA score, MBP, and comorbidities. In model 3, we further adjusted for laboratory results and prescription. This set of covariates was chosen on the bases of their possible influence on ferritin–mortality associations and their influence on the baseline cardiovascular risk of the individuals.

Subgroup analysis was conducted to explore the association between serum ferritin levels and 90-day mortality in different subgroups. Continuous variables should be converted to dichotomous groups according to clinical significance.

The statistical significance was considered at *p* <0.05. Stata 12 was used to conduct the analysis for all data.

## Results

### MIMIC III

After reviewing the data of patients with CHF in the MIMIC III database, a total of 1,739 patients who fulfilled the inclusion criteria were included in the study. The patient selection process is presented in Supplementary Figure S1. The baseline characteristics of all patients were stratified according to 90-day mortality. A total of 1,739 patients were assigned to the survivor (1,270, 73.0%) and non-survivor (469, 27.0%) groups. Compared with survivors, non-survivors were older and more likely to be male. In addition, compared with survivors, non-survivors had a significantly higher SOFA score, Scr, BUN, glucose, potassium, magnesium, sodium, WBC count, and ferritin level. In contrast, the BMI, MBP, hemoglobin, and PLT were significantly lower in non-survivors than in survivors. Patients with AF and AKI had a higher risk of 90-day mortality. The proportion of patients who had used ACEI/ARB within 48 h was significantly lower among the non-survivors than among the survivors. A detailed comparison of the survivors and non-survivors is shown in Table 1. Furthermore, the baseline characteristics of the patients according to their serum ferritin levels are presented in Supplementary Table S1.

**TABLE 1:** Baseline characteristics between survivors and non-survivors in MIMIC III.

Variable	Total (*n* = 1,739)	Survivors (*n* = 1,270)	Non-survivors (*n* = 469)	*p*-value
Age (years)	69 ± 12.9	68.3 ± 13.5	73.6 ± 10.5	***p* <0.001**
Male (*n*%)	875 (50.3)	613 (48.3)	262 (55.9)	***p* = 0.005**
BMI	28.6 ± 7.7	28.9 ± 7.76	27.7 ± 7.38	***p* = 0.003**
**Vital signs**				
MBP (mmHg)	75.4 ± 10.6	76.3 ± 10.9	73.1 ± 9.6	***p* <0.001**
HR (beats/min)	85.3 ± 16.4	85.0 ± 16.5	86.0 ± 16.3	*p* = 0.258
SOFA	5.02 ± 3.1	4.67 ± 2.9	5.97 ± 3.3	***p* <0.001**
**Comorbidities** (*n*%)				
Hypertension	591 (34.0)	454 (35.7)	137 (29.2)	***p* = 0.011**
Diabetes	633 (36.4)	483 (38.0)	150 (32.0)	***p* = 0.020**
AF	735 (42.3)	490 (38.6)	245 (52.2)	***p* <0.001**
AKI	878 (50.5)	596 (46.9)	282 (60.1)	***p* <0.001**
AMI	96 (5.5)	71 (5.6)	25 (5.3)	*p* = 0.833
CKD	522 (30.0)	372 (29.3)	150 (32.0)	*p* = 0.277
COPD	92 (5.3)	60 (4.7)	32 (6.8)	*p* = 0.083
Prior-AMI	148 (8.5)	118 (9.3)	30 (6.4)	*p* = 0.055
**Laboratory results**				
CK (U/L)	155 (257)	158 (258)	148 (254)	*p* = 0.478
Creatinine (mg/dL)	1.8 (2.2)	1.6 (1.9)	2.3 (2.4)	***p* <0.001**
Urea nitrogen (mg/dL)	55.30 ± 34.18	50.70 ± 32.3	67.76 ± 35.97	***p* <0.001**
Glucose (mg/dL)	232.1 ± 128.5	228.24 ± 128.63	242.71 ± 127.85	***p* = 0.037**
Potassium (mEq/L)	5.11 ± 0.8	5.07 ± 0.8	5.25 ± 0.9	***p* = 0.001**
Calcium (mg/dL)	9.2 ± 0.9	9.2 ± 0.8	9.2 ± 1.1	*p* = 0.926
Magnesium (mg/dL)	2.54 ± 0.5	2.52 ± 0.5	2.61 ± 0.5	***p* = 0.001**
Sodium (mEq/L)	143.8 ± 4.62	143.5 ± 4.37	144.7 ± 5.14	***p* <0.001**
WBC (K/UL)	17.5 ± 9.3	16.3 ± 8.1	20.7 ± 11.4	***p* <0.001**
RBC (K/UL)	3.92 ± 0.6	3.94 ± 0.6	3.89 ± 0.6	*p* = 0.089
Hemoglobin (g/dL)	11.6 ± 1.5	11.6 ± 1.5	11.5 ± 1.49	***p* = 0.043**
Platelet (K/UL)	356.5 ± 168.7	365.2 ± 168.4	332.9 ± 167.3	***p* <0.001**
Ferritin (ng/mL)	282 (458)	267 (435)	332 (569)	***p* <0.001**
**Drugs**				
β-blocker	722 (41.5)	541 (42.6)	181 (38.6)	*p* = 0.132
Diuretics	883 (50.8)	659 (51.9)	224 (47.8)	*p* = 0.126
ACEI/ARB	281 (16.2)	236 (18.6)	45 (9.6)	***p* <0.001**

Bold value means *p* value < 0.05.

Table 2 presents the crude outcomes stratified by the serum ferritin groups. The 90-day mortality increased stepwise from level 1 (22.6%) to level 4 (39.6%) (*p* = 0.001). Similarly, the in-hospital and 28-day mortality increased stepwise from group 1 to group 4 (*p* <0.05). Compared with the reference group (70–500 ng/mL), ICU-LOS was longer in the other three groups, especially group 4.

**TABLE 2:** Comparison of outcomes within four ferritin levels in MIMIC III.

Mortality	Ferritin (ng/mL)				*p*-value
	<70 N = 208	≥70 and <500 N = 1007	≥500 and <1000 N = 332	≥1000 N = 192	
In-hospital mortality N (%)	23 (11.1)	124 (12.3)	58 (17.5)	43 (22.4)	*p* <0.001
28-day mortality N (%)	30 (16.8)	142 (19.1)	50 (22.0)	45 (33.9)	*p* = 0.012
90-day mortality N (%)	45 (22.6)	257 (26.5)	93 (30.4)	74 (39.6)	*p* = 0.001
ICU-LOS (days)	3.07 (4.64)	2.62 (4.04)	4.34 (7.59)	4.98 (9.28)	*p* <0.001

**FIGURE 1:**
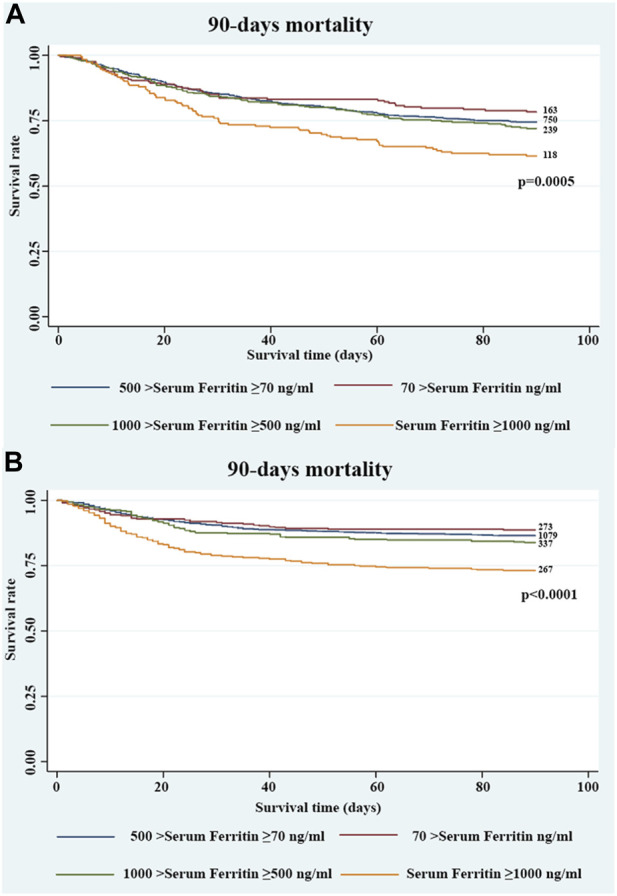
Kaplan–Meier survival curve showing the 90-day mortality stratified by four groups of serum ferritin in critically ill patients with CHF in the **(A)** MIMIC III and **(B)** MIMIC IV databases.

In the extended multivariable logistic models (Table 3), we chose group 2 as the reference group. We observed that for serum ferritin ≥1000 ng/mL, the OR of 90-day mortality was significant in all three adjusted models. In the crude model, serum ferritin ≥1000 ng/mL was associated with an increased 90-day mortality, with the OR increasing stepwise from level 1 (odds ratio [OR], 0.81; 95% confidence interval [CI], 0.57–1.15) to level 4 (OR, 1.81; 95% CI, 1.31–2.50). In the adjusted model 1, patients with serum ferritin ≥1000 ng/mL were more likely to suffer from death within 90 days, with the OR increasing stepwise from level 1 (OR, 0.93; 95% CI, 0.64–1.36) to level 4 (OR, 1.82; 95% CI, 1.28–2.57). Similar results were observed in the adjusted model 2. After adjusting for covariates in model 3, serum ferritin ≥1000 ng/mL continued to be a significant predictor of 90-day mortality (OR, 1.64; 95% CI, 1.13–2.39). Furthermore, serum ferritin ≥1000 ng/mL was also closely associated with increased 28-day mortality (OR, 1.76; 95% CI, 1.14–2.72) (Table 3). After adjusting for covariates in model 3, there was no significant relationship between group 4 and in-hospital mortality (OR, 1.50; 95% CI, 0.94–2.32) (Supplementary Table S2). The results of the Cox regression of 90-day and 28-day mortality were similar (Table 4). The K–M curve showed that serum ferritin ≥1000 ng/mL was associated with the risk of 90-day mortality (Figure 1).

**TABLE 3:** Association between four ferritin levels and mortality in MIMIC III (logistic regression).

Model	Mortality	Ferritin (ng/mL)			
		<70 N = 208	≥70 and <500 N = 1007	≥500 and <1000 N = 332	≥1000 N = 192
Non-adjusted OR (95% CI) *p*-value	28 days	0.86 (0.58–1.28) 0.451	Ref.	1.19 (0.88–1.62) 0.247	2.17(1.55–3.04) <0.001
	90 days	0.81 (0.57–1.15) 0.241	Ref.	1.21 (0.92–1.59) 0.167	1.81(1.31–2.50) <0.001
Model 1 OR (95% CI) *p*-value	28 days	1.19 (0.77–1.85) 0.433	Ref.	1.08 (0.75–1.55) 0.688	1.87(1.25–2.79) 0.002
	90 days	0.93 (0.64–1.36) 0.717	Ref.	1.11 (0.83–1.50) 0.471	1.82(1.28–2.57) 0.001
Model 2 OR (95% CI) *p*-value	28 days	1.19 (0.76–1.85) 0.447	Ref.	1.06 (0.74–1.53) 0.752	1.91(1.28–2.86) 0.002
	90 days	0.93 (0.64–1.36) 0.725	Ref.	1.09 (0.81–1.46) 0.573	1.84(1.29–2.62) 0.001
Model 3 OR (95% CI) *p*-value	28 days	1.24 (0.79–1.96) 0.355	Ref.	0.97 (0.66–1.42) 0.861	1.76(1.14–2.72) 0.011
	90 days	1.01 (0.69–1.49) 0.946	Ref.	0.97 (0.71–1.32) 0.837	1.64(1.13–2.39) 0.009

**TABLE 4:** Association between four ferritin levels and mortality in MIMIC III (Cox regression).

Model	Mortality	Ferritin (ng/mL)			
		<70 N = 208	≥70 and <500 N = 1007	≥500 and <1000 N = 332	≥1000 N = 192
Non-adjusted HR (95% CI) *p*-value	28 days	01.03 (0.70–1.53) 0.878	Ref.	1.07 (0.78–1.48) 0.658	1.73(1.24–2.42) 0.001
	90 days	0.84 (0.61–1.15) 0.281	Ref.	1.11 (0.87–1.40) 0.401	1.65(1.27–2.13) 0.000
Model 1 HR (95% CI) *p*-value	28 days	1.16 (0.78–1.73) 0.457	Ref.	1.07 (0.77–1.49) 0.668	1.71(1.22–2.41) 0.002
	90 days	0.94 (0.68–1.29) 0.701	Ref.	1.09 (0.86–1.39) 0.475	1.64(1.26–2.14) 0.000
Model 2 HR (95% CI) *p*-value	28 days	1.16 (0.78–1.72) 0.474	Ref.	1.07 (0.77–1.48) 0.699	1.76(1.25–2.47) 0.001
	90 days	0.94 (0.68–1.29) 0.692	Ref.	1.08 (0.85–1.38) 0.516	1.67(1.28–2.18) 0.000
Model 3 HR (95% CI) *p*-value	28 days	1.16 (0.77–1.74) 0.473	Ref.	1.00 (0.72–1.40) 0.980	1.70(1.19–2.43) 0.003
	90 days	0.97 (0.70–1.34) 0.852	Ref.	1.00 (0.78–1.28) 0.986	1.55(1.18–2.03) 0.002

In addition, we explored the association between serum ferritin levels and long-term (1- and 5-year) mortality, and the results showed that the serum ferritin level was not a predictor of long-term mortality after adjusting for multiple covariates (Supplementary Table S3). However, compared with the reference group, serum ferritin ≥500 ng/mL significantly increased the ICU-LOS in all three adjusted models (Supplementary Table S4). Furthermore, the serum ferritin level was not associated with the risk of readmission within 30 days or 1 year of discharge (Supplementary Table S5).

We subsequently conducted subgroup analyses to evaluate the relationship between serum ferritin levels and 90-day mortality in different subgroups, and no significant interaction was detected (Supplementary Table S6).

### MIMIC IV

To validate the results using a larger database, we also explored the MIMIC IV database. After reviewing the data of patients with CHF in the MIMIC IV database, 2,322 patients who fulfilled the inclusion criteria were included in the study. Detailed baseline characteristics of all patients are shown in Supplementary Table S7. In the extended multivariable logistic models (Table 5), we observed that for serum ferritin levels ≥1000 ng/mL, the ORs of in-hospital (OR, 1.70; 95% CI, 1.18–2.46), 28-day (OR, 1.83; 95% CI, 1.24–2.69), and 90-day mortality (OR, 1.57; 95% CI, 1.11–2.22) were significant in the adjusted model, with the OR increasing stepwise from group 1 to group 4. The K–M curve revealed similar results (*p* <0.0001) (Figure 1).

**TABLE 5:** Association between four ferritin levels and mortality in MIMIC IV (logistic regression).

Model	Mortality	Ferritin (ng/mL)			
		<70 N = 308	≥70 and <500 N = 1247	≥500 and <1000 N = 402	≥1000 N = 365
Adjusted	In-hospital	0.92 (0.55–1.51) 0.729	Ref.	1.10 (0.75–1.61) 0.635	1.70(1.18–2.46) 0.005
OR (95% CI)	28 days	0.91 (0.55–1.53) 0.732	Ref.	1.32 (0.89–1.97) 0.171	1.83(1.24–2.69) 0.002
*p*-value	90 days	0.96 (0.61–1.50) 0.843	Ref.	1.08 (0.76–1.54) 0.675	1.57(1.11–2.22) 0.011

## Discussion

In this study, we observed that a high serum ferritin level (≥1000 ng/mL) was significantly associated with high risks, in terms of adjusted 90-day mortality as well as high 28-day mortality, among patients with CHF. In addition, in the MIMIC IV group, patients with CHF with high serum ferritin levels tended to have a higher risk of in-hospital mortality, which was different from the result of the MIMIC III group. Furthermore, serum ferritin levels appeared to increase ICU-LOS; however, no group differences in the long-term mortality or readmission rates were observed.

High serum ferritin levels are closely associated with a series of cardiovascular diseases, especially HF. Previous studies pointed out that individuals with serum ferritin levels >2500 ng/mL were more likely to develop HF (KIRK et al., 2009). In a community-based study, the risk of HF was found to be significantly higher in the group with an average ferritin concentration of 358 ng/mL (SILVESTRE et al., 2017). This finding is consistent with those of a large prospective study and our study (KLIP et al., 2017). In our study, serum ferritin levels >1000 ng/mL indicated a higher risk of short-term mortality among patients with CHF, and three models were selected to adjust for confounding factors. Although there was no relationship between high serum ferritin levels and in-hospital mortality, the association was significant in the MIMIC IV group. In addition, we observed that the OR of short-term mortality in the MIMIC IV group increased stepwise from ferritin level 1 to level 4, which was not observed in the MIMIC III group. We believe that this is due to the larger sample size of the MIMIC IV database than that of the MIMIC III database.

The bone marrow iron content and liver iron content are gold standards for the evaluation of iron deficiency and overload, respectively. However, these tests are invasive and expensive, thereby limiting their use in routine practice. Serum ferritin levels are considered to reflect the iron status. Low ferritin levels indicate iron deficiency, whereas increased ferritin levels indicate an iron overload. In the present analysis, the highest iron level was observed in the group with serum ferritin ≥1000 ng/mL. Toxicity induced by excessive iron causes significant damage to various tissues and organs (FLEMING and PONKA, 2012). More importantly, cardiac tissues are vulnerable to free iron accumulation (GUJJA et al., 2010). Free iron can be taken up by cardiomyocytes, and it can participate in intracellular ROS production via Fenton’s reaction (CHAN et al., 2015; BABA et al., 2018). Furthermore, excessive free iron can enter the mitochondria and generate mitochondrial oxidative stress, resulting in the impaired mitochondrial function characterized by decreased mitochondrial respiration and membrane potential depolarization (KUMFU et al., 2012; CHEN et al., 2014; SRIPETCHWANDEE et al., 2014). Therefore, iron overload, characterized by serum ferritin levels >1000 ng/mL, may contribute to a higher risk of mortality. However, it was difficult to rule out the influence of inflammation in our study.

Ferritin is also an acute-phase protein, and elevated ferritin levels can be present in an inflammatory context. In order to adjust for inflammatory factors, we attempted to extract the data on the C-reactive protein (CRP) level and erythrocyte sedimentation rate (ESR). However, we did not include these covariates because the data contained approximately 70% missing values. Therefore, it is likely that residual confounding may persist, given the high chance of ferritin being associated with cardiometabolic risks on the basis of its biological roles in pleiotropic influences other than iron metabolism (SUÁREZ-ORTEGÓN et al., 2022). The most commonly used definition of iron deficiency is ferritin levels <100 ng/mL or 100–300 ng/mL and a transferrin saturation <20%; however, GROTE BEVERBORG et al. (2018) reported that 28% false positives would be observed if these criteria are used. All these suggest a necessity for a correction of cutoff values of ferritin levels that indicated the iron status for HF patients compared to the general population. According to the new World Health Organization (WHO) guide, ferritin levels <70 ng/mL was defined as iron deficiency in individuals with infection or inflammation, and non-healthy individuals with ferritin levels >500 ng/mL were defined as having a risk of iron overload (WHO Guidelines Approved by the Guidelines Review Committee [M], 2020). Based on this guide and previous study (GROTE BEVERBORG et al., 2018), group 2 (ferritin ≥70 and <500 ng/mL) was selected as the reference group. We further set up group 4 (ferritin ≥1000 ng/mL) for more accurate results. However, the criteria for iron deficiency and overload in patients with HF still needed to be addressed. Even though we believe that serum ferritin ≥1000 ng/mL is an independent predictor of short-term mortality in ICU patients with CHF, the extent to which increased ferritin levels truly reflect iron stores is unclear.

Over the years, the identification of patients at a risk for mortality due to CHF using biomarkers alongside clinical characteristics has gained considerable interest. Serum ferritin is a widely available routine marker, and it is relatively inexpensive to measure and may provide additional information in terms of etiology, clinical risk, and disease severity. However, in clinical practice, serum ferritin levels are not routinely measured in patients with HF. In addition, previous studies have mostly focused on the relationship between iron deficiency and HF. The DAPA-HF trial defined serum ferritin levels <100 ng/mL as iron deficiency and concluded that HF patients with ferritin levels <100 ng/mL had worse outcomes than those with ferritin levels >100 ng/mL (DOCHERTY et al., 2022). The IRONMAN trial supported the benefit of iron repletion in the population (KALRA et al., 2022). Most studies divided patients into iron deficiency (ferritin <100 ng/mL) and non-iron deficiency and did not further stratify patients with ferritin levels >100 ng/mL, thus not detecting the relationship between high ferritin levels and mortality in patients with CHF. According to our study, physicians should note that patients with CHF with high serum ferritin levels might be at a high risk of mortality, and this conclusion can be applied when physicians make decisions regarding therapeutic approaches at the initial admission of critically ill patients. Thus, integrating ferritin in existing or future clinical models for risk stratification might increase the prognostic performance by delineating high-risk patients who could benefit from closer monitoring. Finally, whether different approaches to reduce ferritin serum levels would decrease the mortality risk of HF opens new avenues for further studies.

## Strengths and limitations

The study has several strengths. First, this is the first study to explore the relationship between serum ferritin levels and short-term mortality in patients with CHF in the ICU. Second, the analysis was adjusted for potential confounding factors that might affect the conclusions, and the results were confirmed through repeating the analysis using a second database, which had a different sample size from the first. The limitations of the study should also be addressed. First, this study was a single-center study, and the conclusions might differ when using patients’ records from other centers. Therefore, a larger prospective multicenter study is required. Second, the data were obtained from an online database and were not validated with data from our hospital. Third, for patients with CHF who had been admitted to the ICU on multiple occasions, only data regarding their initial admission were analyzed, which posed a potential selection bias. Fourth, although we have adjusted for as many covariate variables as possible to diminish the possible influences, owing to the retrospective design of the study, residual confounding may exist, which needs to be investigated in the future. Finally, some parameters, such as the ejection fraction and CRP level, were not assessed in the multivariate analysis because of the excessive proportion of missing data.

## Conclusion

Serum ferritin ≥1000 ng/mL is an independent predictor of short-term mortality in ICU patients with CHF, including in-hospital, 28-day, and 90-day mortality.

## Data Availability

The original contributions presented in the study are included in the article/Supplementary Material; further inquiries can be directed to the corresponding authors.

## Abbreviations

AKI, acute kidney injury; AMI, acute myocardial infarction; ACEI/ARB, angiotensin-converting enzyme inhibitor/angiotonin receptor blocker; AF, atrial fibrillation; BUN, blood urea nitrogen; BMI, body mass index; CHF, chronic heart failure; CI, confidence interval; CK, creatine kinase; CKD, chronic kidney disease; COPD, chronic obstructive pulmonary disease; CRP, C-reactive protein; ESR, erythrocyte sedimentation rate; HF, heart failure; HR, heart rate; Hb, hemoglobin; ICD, International Classification of Diseases; ICU, intensive care unit; ICU-LOS, ICU length of stay; IQR, interquartile range; K–M, Kaplan–Meier; MBP, mean blood pressure; MIMIC, Multiparameter Intelligent Monitoring in Intensive Care; OR, odds ratio; PLT, platelet; RBC, red blood cell; SOFA, Sequential Organ Failure Assessment; Scr, serum creatinine; STROBE, STrengthening the Reporting of OBservational studies in Epidemiology; WBC, white blood cell.
