# Supplementary material for: Serum ferritin and the risk of short-term mortality in critically ill patients with chronic heart failure: a retrospective cohort study
Source: Front Physiol. 2023 Jul 13;14:1148891. doi: 10.3389/fphys.2023.1148891 (PMC10372222; doi:10.3389/fphys.2023.1148891)

**Serum ferritin and risk of short-term mortality in critically ill patients with chronic heart failure: A retrospective cohort study**

Zijing Zhou^1^, MD, Deyi Yang^2^, MD, Chan Li^3^, MD, Ting Wu^3#^, MD, Ruizheng Shi^3#^, MD

1 Department of Cardiovascular Medicine, The Third Xiangya Hospital, Central South University, Changsha, Hunan, China

2 Department of Gastroenterology, Beijing Friendship Hospital, Capital Medical University, Beijing, China

3 Department of Cardiovascular Medicine, Xiangya Hospital, Central South University, Changsha, Hunan, China

#Corresponding author: Ting Wu, wuting127039@163.com

Ruizheng Shi, [xyshiruizheng@csu.edu.cn](mailto:xyshiruizheng@csu.edu.cn)

**eTable 1 Baseline characteristics according to serum ferritin levels**

| **Variable** | **Ferritin(ng/ml)** | | | | ***P* value** |
| --- | --- | --- | --- | --- | --- |
|  | <70 | ≥70;<500 | ≥500;<100 | ≥1000 |  |
|  | N=208 | N=1007 | N=332 | N=192 |  |
| Age | 70.2 ± 12.8 | 70.6 ± 12.7 | 68.5 ± 12.5 | 66.9 ± 14.4 | < 0.001 |
| Male | 77 (37) | 483 (48) | 195 (58.7) | 120 (62.5) | < 0.001 |
| BMI | 29.6 ± 9.0 | 28.4 ± 7.5 | 28.6 ± 7.8 | 28.6 ± 6.7 | 0.204 |
| **Vital signs** |  |  |  |  |  |
| MBP | 101.0 ± 24.9 | 103.5 ± 28.2 | 104.2 ± 29.9 | 107.5 ± 31.7 | 0.143 |
| HR | 101.3 ± 21.3 | 102.3 ± 21.6 | 106.0 ± 22.3 | 109.1 ± 22.0 | < 0.001 |
| SOFA | 4.0 ± 2.4 | 4.8 ± 2.9 | 5.6 ± 3.4 | 6.2 ± 3.4 | < 0.001 |
| **Comorbidities** |  |  |  |  |  |
| Hypertension | 76 (36.5) | 356 (35.4) | 104 (31.3) | 55 (28.6) | 0.177 |
| Diabetes | 82 (39.4) | 366 (36.3) | 110 (33.1) | 75 (39.1) | 0.401 |
| AF | 100 (48.1) | 425 (42.2) | 137 (41.3) | 73 (38) | 0.218 |
| AKI | 87 (41.8) | 493 (49) | 189 (56.9) | 109 (56.8) | 0.001 |
| AMI | 8 (3.8) | 60 (6) | 21 (6.3) | 7 (3.6) | 0.363 |
| CKD | 58 (27.9) | 296 (29.4) | 95 (28.6) | 73 (38) | 0.078 |
| COPD | 17 (8.2) | 54 (5.4) | 16 (4.8) | 5 (2.6) | 0.095 |
| Prio-AMI | 21 (10.1) | 95 (9.4) | 23 (6.9) | 9 (4.7) | 0.092 |
| **Laboratory results** |  |  |  |  |  |
| Urea nitrogen | 44.3 ± 28.1 | 53.2 ± 33.2 | 62.0 ± 36.7 | 66.9 ± 35.7 | < 0.001 |
| Glucose | 202.8 ± 95.9 | 229.2 ± 125.0 | 244.2 ± 141.4 | 258.6 ± 146.7 | < 0.001 |
| Potassium | 4.9 ± 0.7 | 5.1 ± 0.9 | 5.3 ± 0.8 | 5.2 ± 0.9 | < 0.001 |
| Calcium | 9.2 ± 0.8 | 9.2 ± 0.8 | 9.2 ± 1.0 | 9.4 ± 1.3 | 0.011 |
| Magnesium | 2.4 ± 0.3 | 2.5 ± 0.6 | 2.6 ± 0.5 | 2.6 ± 0.6 | < 0.001 |
| Sodium | 142.8 ± 3.8 | 143.6 ± 4.5 | 144.4 ± 5.2 | 145.0 ± 4.8 | < 0.001 |
| WBC | 14.1 ± 6.0 | 16.8 ± 8.8 | 20.0 ± 10.6 | 20.6 ± 10.9 | < 0.001 |
| RBC | 4.1 ± 0.6 | 3.9 ± 0.6 | 3.9 ± 0.5 | 3.9 ± 0.6 | < 0.001 |
| Hemoglobin | 11.2 ± 1.5 | 11.6 ± 1.5 | 11.6 ± 1.5 | 11.7 ± 1.7 | < 0.001 |
| Platelet | 324.9 ± 133.6 | 350.7 ± 162.5 | 393.8 ± 189.5 | 357.1 ± 185.6 | < 0.001 |
| CK | 91.0 (56.0, 277.3) | 166.0 (69.0, 328.0) | 147.0 (55.8, 319.0) | 216.5 (72.8, 391.8) | < 0.001 |
| Creatinine | 1.4 (1.0, 2.2) | 1.7 (1.1, 3.0) | 2.2 (1.3, 4.1) | 2.8 (1.4, 5.2) | < 0.001 |
| Ferritin | 39.5 (28.0, 55.0) | 221.0 (131.0, 329.0) | 687.5 (577.8, 820.2) | 1396.0 (1167.0, 1638.0) | < 0.001 |
| Iron | 29.0 (20.0, 46.2) | 35.0 (22.5, 52.0) | 34.0 (23.0, 54.2) | 40.0 (26.0, 67.2) | < 0.001 |
| **Drugs** |  |  |  |  |  |
| β-blocker | 88 (42.3) | 428 (42.5) | 132 (39.8) | 74 (38.5) | 0.664 |
| Diuretics | 122 (58.7) | 521 (51.7) | 158 (47.6) | 82 (42.7) | 0.008 |
| ACEI/ARB | 46 (22.1) | 175 (17.4) | 37 (11.1) | 23 (12) | 0.002 |

**eTable 2 Association between four ferritin levels and in-hospital mortality in MIMIC-III (Logistic regression)**

| **Models** | **Mortality** | **Ferritin(ng/ml)** | | | |
| --- | --- | --- | --- | --- | --- |
|  |  | <70  N=208 | ≥70;<500  N=1007 | ≥500;<1000  N=332 | ≥1000  N=192 |
| Non-adjusted  OR (95% CI)  *P*-Value | In-hospital | 0.89(0.55-1.42) 0.613 | ref | 1.51(1.07-2.12) 0.018 | **2.06(1.39-3.03)**  **<0.001** |
| Model 1  OR (95% CI)  *P*-Value | In-hospital | 1.04(0.64-1.69) 0.862 | ref | 1.41(0.99-2.01) 0.059 | **1.82(1.21-2.74) 0.004** |
| Model 2  OR (95% CI)  *P*-Value | In-hospital | 1.05(0.64-1.71) 0.844 | ref | 1.39(0.96-1.97) 0.079 | **1.87(1.24-2.83) 0.003** |
| Model 3  OR (95% CI)  *P*-Value | In-hospital | 1.29(0.77-2.16) 0.775 | ref | 1.19(0.80-1.75) 0.389 | 1.50(0.94-2.32) 0.089 |

**eTable 3 Association between four ferritin levels and long-term mortality in MIMIC-III (Logistic regression)**

| **Models** | **Mortality** | **Ferritin(ng/ml)** | | | |
| --- | --- | --- | --- | --- | --- |
|  |  | <70  N=208 | ≥70;<500  N=1007 | ≥500;<1000  N=332 | ≥1000  N=192 |
| Non-adjusted  OR (95% CI)  *P*-Value | 1 Year | 0.82(0.60-1.12) 0.214 | ref | 1.14(0.89-1.47) 0.296 | **1.46(1.07-1.98)**  **0.018** |
|  | 5 Year | 0.82(0.61-1.12) 0.200 | ref | 0.99(0.76-1.28) 0.985 | 1.22(0.89-1.68)  0.213 |
| Model 1  OR (95% CI)  *P*-Value | 1 Year | 0.94(0.67-1.30) 0.688 | ref | 1.13(0.87-1.47) 0.366 | **1.45(1.04-2.02) 0.028** |
|  | 5 Year | 0.90(0.66-1.23) 0.522 | ref | 1.02(0.78-1.34) 0.852 | 1.33(0.94-1.87)  0.106 |
| Model 2  OR (95% CI)  *P-*Value | 1 Year | 0.93(0.67-1.29) 0.659 | ref | 1.10(0.84-1.44) 0.476 | **1.46(1.04-2.04) 0.028** |
|  | 5 Year | 0.88(0.64-1.21) 0.436 | ref | 0.99(0.76-1.31) 0.991 | 1.33(0.94-1.89)  0.107 |
| Model 3  OR (95% CI)  *P*-Value | 1 Year | 0.98(0.69-1.37) 0.889 | ref | 0.99(0.75-1.32) 0.995 | 1.32(0.93-1.88) 0.126 |
|  | 5 Year | 0.90(0.65-1.25) 0.527 | ref | 0.92(0.70-1.22) 0.579 | 1.22(0.85-1.75)  0.291 |

**eTable 4 Association between four ferritin levels and ICU-LOS in MIMIC-III**

| **Models** | **Outcomes** | **Ferritin(ng/ml)** | | | |
| --- | --- | --- | --- | --- | --- |
|  |  | <70  N=208 | ≥70;<500  N=1007 | ≥500;<1000  N=332 | ≥1000  N=192 |
| Non-adjusted  Coef (95% CI)  *P*-Value | ICU-LOS | **-1.48(-2.86- -0.10)**  **0.035** | ref | **3.11(1.97-4.26) 0.000** | **3.63(2.21-5.06)**  **0.000** |
| Model 1  Coef (95% CI)  *P*-Value | ICU-LOS | -0.95(-2.29- -0.38) 0.162 | ref | **2.49(1.38-3.60) 0.000** | **2.46(1.07-3.85) 0.001** |
| Model 2  Coef (95% CI)  *P*-Value | ICU-LOS | -1.01(-2.34-0.32) 0.136 | ref | **2.42(1.31-3.52) 0.000** | **2.46(1.07-3.85) 0.001** |
| Model 3  Coef (95% CI)  *P*-Value | ICU-LOS | 0.11(-1.11-1.34) 0.856 | ref | **1.43(0.40-2.45) 0.006** | **1.42(0.15-2.70) 0.029** |

**eTable 5 Association between four ferritin levels and the rate of readmission (30 days and 1 year) in MIMIC-III (Logistic regression)**

| **Models** | **Readmission** | **Ferritin(ng/ml)** | | | |
| --- | --- | --- | --- | --- | --- |
|  |  | <70  N=208 | ≥70;<500  N=1007 | ≥500;<1000  N=332 | ≥1000  N=192 |
| Non-adjusted  OR (95% CI)  *P*-Value | 30 days | 0.92(0.51-1.67)  0.792 | ref | 1.04(0.65-1.67) 0.865 | 1.49(0.88-2.50)  0.135 |
|  | 1 Year | 0.98(0.66-1.44) 0.919 | ref | 0.97(0.70-1.33) 0.839 | 1.19(0.81-1.74)  0.368 |
| Model 1  OR (95% CI)  *P*-Value | 30 days | 0.95(0.52-1.73) 0.872 | ref | 1.01(0.63-1.64) 0.940 | 1.45(0.85-2.47) 0.170 |
|  | 1 Year | 0.97(0.66-1.43) 0.889 | ref | 0.98(0.71-1.35) 0.889 | 1.21(0.82-1.78)  0.339 |
| Model 2  OR (95% CI)  *P*-Value | 30 days | 0.93(0.51-1.70) 0.827 | ref | 1.00(0.62-1.62) 0.977 | 1.45(0.85-2.47)  0.176 |
|  | 1 Year | 0.96(0.65-1.41) 0.822 | ref | 0.97(0.70-1.35) 0.872 | 1.20(0.81-1.77)  0.360 |
| Model 3  OR (95% CI)  *P*-Value | 30 days | 0.86(0.47-1.57) 0.619 | ref | 1.00(0.62-1.63) 0.984 | 1.52(0.88-2.63) 0.131 |
|  | 1 Year | 0.90(0.61-1.34) 0.619 | ref | 0.97(0.69-1.35) 0.845 | 1.22(0.82-1.81)  0.327 |

**eTable 6 Association between four ferritin levels and 90-day mortality in MIMIC-III in subgroups**

| **Subgroups** | **Ferritin(ng/ml)** | | | | |  |
| --- | --- | --- | --- | --- | --- | --- |
|  | <70  N=208 | | ≥70;<500  N=1007 | ≥500;<1000  N=332 | ≥1000  N=192 | *P* for interaction |
| **Gender** [OR (95% CI); *P*-Value] | | | | | | 0.467 |
| Female | 0.70(0.40-1.21); 0.202 | | ref | 0.91(0.56-1.48); 0.713 | 1.55(0.86-2.79); 0.148 |  |
| Male | 1.52(0.86-2.72); 0.153 | | ref | 1.03(0.67-1.59); 0.878 | 1.85(1.116-3.06); 0.017 |  |
| **Age, years** | |  |  |  |  |  |
| ≤72 | 1.00(0.63-1.60); 0.971 | | ref | 1.16(0.78-1.72); 0.457 | 2.41(1.42-4.08); 0.001 | 0.066 |
| >72 | 1.02(0.59-1.77); 0.938 | | ref | 1.23(0.79-1.92); 0.357 | 2.76(1.57-4.86); <0.001 |  |
| **BMI** |  | |  |  |  | 0.955 |
| ≤25 | 1.05(0.55-2.00); 0.879 | | ref | 1.00(0.59-1.70); 1.00 | 1.62(0.82-3.2); 0.167 |  |
| >25 | 0.99(0.60-1.62); 0.961 | | ref | 0.93(0.62-1.34); 0.707 | 1.66(1.05-2.62); 0.031 |  |
| **MBP** |  | |  |  |  |  |
| ≤70 | 0.80(0.40-1.58); 0.515 | | ref | 1.43(0.84-2.44); 0.185 | 2.61(1.34-5.11); 0.005 | 0.06 |
| >70 | 1.17(0.73-1.89); 0.517 | | ref | 0.77(0.51-1.15); 0.197 | 1.30(0.81-2.09); 0.282 |  |
| **Hypertension** |  | |  |  |  |  |
| No | 1.07(0.67-1.73); 0.770 | | ref | 1.01(0.70-1.47); 0.951 | 1.87(1.22-2.88); 0.004 | 0.421 |
| Yes | 0.76(0.38-1.53); 0.442 | | ref | 0.71(0.39-1.30); 0.269 | 0.93(0.38-2.26); 0.870 |  |
| **Diabetes** |  | |  |  |  |  |
| No | 0.87(0.53-1.43); 0.589 | | ref | 0.82(0.55-1.21); 0.318 | 1.50(0.92-2.44); 0.103 | 0.545 |
| Yes | 1.30(0.69-2.47); 0.418 | | ref | 1.29(0.75-2.23); 0.355 | 2.15(1.16-3.98); 0.015 |  |
| **AF** |  | |  |  |  | 0.573 |
| No | 1.09(0.61-1.95); 0.761 | | ref | 0.74(0.47-1.17); 0.196 | 1.35(0.81-2.24); 0.243 |  |
| Yes | 0.94(0.55-1.59); 0.811 | | ref | 1.28(0.82-2.00); 0.272 | 2.06(1.15-3.68); 0.015 |  |
| **AKI** |  | |  |  |  | 0.998 |
| No | 1.04(0.60-1.81); 0.878 | | ref | 0.91(0.55-1.49); 0.697 | 1.33(0.72-2.47); 0.365 |  |
| Yes | 1.03(0.59-1.81); 0.915 | | ref | 0.98(0.65-1.47); 0.908 | 1.65(1.01-2.69); 0.044 |  |
| **Creatinine** |  | |  |  |  | 0.614 |
| ≤1.2 | 1.3(0.60-2.77); 0.519 | | ref | 0.77(0.34-1.76); 0.536 | 2.78(1.04-7.42); 0.042 |  |
| >1.2 | 0.94(0.59-1.49); 0.783 | | ref | 0.97(0.69-1.36); 0.846 | 1.40(0.93-2.10); 0.105 |  |
| **Urea nitrogen** |  | |  |  |  | 0.219 |
| ≤21 | 3.05(0.82-11.3); 0.096 | | ref | 0.29(0.03-3.09); 0.305 | 0.45(0.01-21.6); 0.685 |  |
| >21 | 0.93(0.61-1.41); 0.737 | | ref | 0.98(0.72-1.35); 0.923 | 1.70(1.17-2.48); 0.006 |  |
| **Glucose** |  | |  |  |  | 0.672 |
| ≤126 | 2.48(0.54-11.4); 0.243 | | ref | 2.14(0.30-15.3); 0.449 | 2.80(0.15-53.5); 0.493 |  |
| >126 | 0.95(0.63-1.43); 0.816 | | ref | 0.93(0.67-1.28); 0.648 | 1.59(1.08-2.33); 0.019 |  |
| **Potassium** |  | |  |  |  | 0.595 |
| ≤4.9 | 0.76(0.42-1.39); 0.375 | | ref | 0.99(0.56-1.73); 0.967 | 1.67(0.89-3.12); 0.112 |  |
| >4.9 | 1.24(0.73-2.11); 0.431 | | ref | 0.94(0.64-1.38); 0.737 | 1.69(1.05-2.72); 0.032 |  |
| **Magnesium** |  | |  |  |  | 0.423 |
| ≤2.2 | 1.10(0.38-3.24); 0.856 | | ref | 1.93(0.70-5.32); 0.205 | 1.45(0.21-9.86); 0.707 |  |
| >2.2 | 1.03(0.67-1.58); 0.883 | | ref | 0.86(0.61-1.20); 0.365 | 1.59(1.08-2.34); 0.018 |  |
| **Sodium** |  | |  |  |  | 0.421 |
| ≤145 | 1.07(0.68-1.69); 0.778 | | ref | 1.07(0.70-1.62); 0.757 | 1.99(1.21-3.28); 0.007 |  |
| >145 | 0.94(0.43-2.06); 0.870 | | ref | 0.79(0.49-1.29); 0.356 | 1.24(0.69-2.22); 0.468 |  |
| **WBC** |  | |  |  |  | 0.875 |
| ≤10 | 1.24(0.50-3.09); 0.642 | | ref | 1.28(0.46-3.53); 0.636 | 0.91(0.18-4.69); 0.912 |  |
| >10 | 0.95(0.61-1.48); 0.826 | | ref | 1.00(0.72-1.39); 0.996 | 1.77(1.21-2.60); 0.003 |  |
| **Hemoglobin** |  | |  |  |  | 0.969 |
| ≤11 | 0.90(0.51-1.59); 0.710 | | ref | 1.06(0.63-1.77); 0.827 | 1.98(1.08-3.63); 0.027 |  |
| >11 | 1.14(0.73-1.79); 0.567 | | ref | 0.97(0.69-1.36); 0.849 | 1.43(0.94-2.17); 0.091 |  |
| **Platelet** |  | |  |  |  |  |
| ≤300 | 1.02(0.59-1.79); 0.937 | | ref | 1.31(0.80-2.16); 0.280 | 3.18(1.77-5.73); <0.001 | 0.080 |
| >300 | 1.00(0.58-1.76); 0.982 | | ref | 0.75(0.50-1.13); 0.172 | 0.97(0.57-1.64); 0.908 |  |
| **ACEI/ARB** |  | |  |  |  | 0.725 |
| No | 1.05(0.69-1.61); 0.816 | | ref | 0.95(0.69-1.33); 0.781 | 1.72(1.16-2.55); 0.007 |  |
| Yes | 0.74(0.25-2.19); 0.581 | | ref | 1.04(0.30-3.57); 0.953 | 0.85(0.18-4.04); 0.837 |  |

**eTable 7 Baseline characteristics between survivors and non-survivors in MIMIC-IV**

| **Variable** | **Total**  **(n=2322)** | **Survivors**  **(n=1956)** | **Non-survivors**  **(n=366)** | ***P* Value** |
| --- | --- | --- | --- | --- |
| Age(years) | 70.06±13.8 | 69.6±13.9 | 72.6±12.9 | ***P*<0.001** |
| Male(n%) | 1269(50.3) | 1056() | 213() | *P*=0.138 |
| BMI | 29.4±8.03 | 29.6±8.12 | 28.6±7.53 | ***P*=0.039** |
| **Vital signs** | | | | |
| MBP (mmHg) | 75.4±10.9 | 75.97±11.0 | 72.2±9.8 | ***P*<0.001** |
| HR (beats/min) | 85.6±16.8 | 85.2±16.6 | 88.0±17.4 | ***P*=0.003** |
| SOFA | 6.08±3.76 | 5.58±3.5 | 8.80±4.03 | ***P*<0.001** |
| **Comorbidities** | | | | |
| Hypertension | 528 | 461 | 67 | ***P*=0.027** |
| Diabetes | 556 | 479 | 77 | *P*=0.156 |
| AF | 1150 | 942 | 208 | ***P*=0.002** |
| AKI | 1391 | 1124 | 267 | ***P*<0.001** |
| AMI | 243 | 195 | 48 | *P*=0.071 |
| CKD | 1073 | 886 | 187 | ***P*=0.041** |
| COPD | 274 | 215 | 59 | ***P*=0.005** |
| Prio-AMI | 312 | 260 | 52 | *P*=0.637 |
| **Laboratory results** | | | | |
| CK (U/L) | 127(306) | 124(277) | 149.5(462.2) | *P*=0.128 |
| Creatinine(mg/dl) | 1.9(2.3) | 1.8(2.1) | 2.7(2.6) | ***P*<0.001** |
| Urea nitrogen(mg/dl) | 57.9±35.6 | 55.2±34.6 | 72.52±37.7 | ***P*<0.001** |
| Glucose(mg/dl) | 246.6±156.7 | 239.4±149.8 | 285.4±184.7 | ***P*<0.001** |
| Potassium(mEq/L) | 5.28±0.97 | 5.24±0.96 | 5.52±1.00 | ***P*<0.001** |
| Calcium(mg/dl) | 9.3±0.9 | 9.3±0.8 | 9.4±1.2 | ***P*<0.001** |
| Magnesium(mg/dl) | 2.65±0.7 | 2.62±0.6 | 2.79±0.9 | ***P*<0.001** |
| Sodium(mEq/L) | 143.6±4.9 | 143.4±4.74 | 144.3±5.75 | ***P*<0.001** |
| WBC(K/UL) | 17.1±9.6 | 16.2±8.9 | 22.2±11.9 | ***P*<0.001** |
| RBC(K/UL) | 3.80±0.70 | 3.82±0.7 | 3.69±0.7 | ***P*=0.002** |
| Hemoglobin (g/dl) | 11.0±1.9 | 11.1±2.0 | 10.8±1.8 | ***P*=0.011** |
| Platelet(K/UL) | 318.3±154.1 | 322.9±152.7 | 293.6±159.5 | ***P*<0.001** |
| Ferritin(ng/ml) | 288(545) | 271(494) | 417(902) | ***P*<0.001** |
| Iron(ug/dl) | 36(34) | 36(32) | 33(41) | *P*=0.747 |
| **Drugs** | | | | |
| Β-blocker | 1352 | 1157 | 195 | ***P*=0.037** |
| Diuretics | 1312 | 1109 | 203 | *P*=0.662 |
| ACEI/ARB | 990 | 909 | 81 | ***P*<0.001** |

**eFigure 1 Flowchart of patient selection in MIMIC-III**


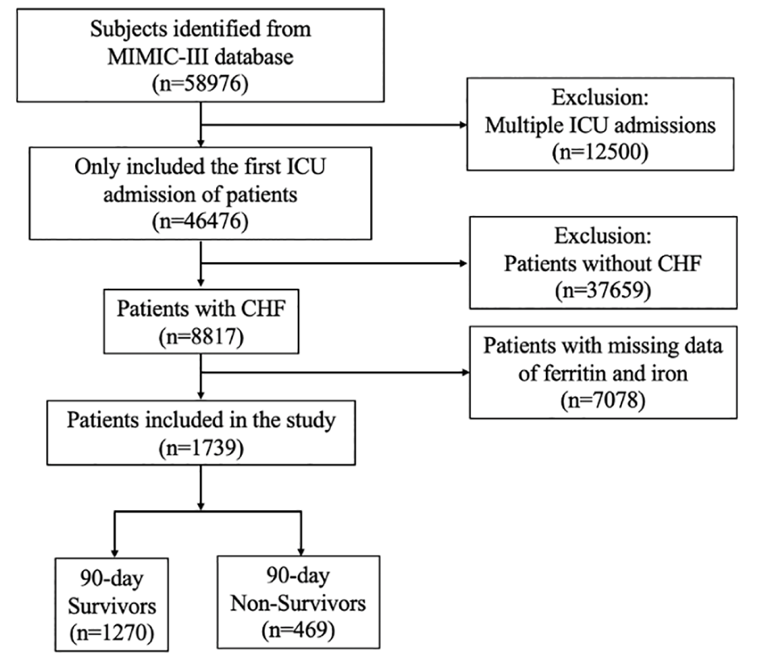

Supplement: Supplementary file 1 [file DataSheet1.docx]
